# The complete chloroplast genome sequence of *Illigera celebica*

**DOI:** 10.1080/23802359.2020.1778562

**Published:** 2020-06-16

**Authors:** Yaxuan Xin, Jing Xin, Guoqiong Yao, Yaya Qu, Fayu Feng, Yu Song, Zhenghai Sun

**Affiliations:** a South and Southeast Asia Joint R&D Center of Economic Forest Full Industry Chain of Yunnan Province, Southwest Forestry University, Kunming, PR China; b International Technologial Cooperation Base of High Effective Economic Forestry cultivating of Yunnan Province, Southwest Forestry University, Kunming, PR China; c Center for Integrative Conservation, Xishuangbanna Tropical Botanical Garden, Chinese Academy of Sciences, Jinghong, PR China

**Keywords:** *Illigera;* genome, phylogenetic relationship

## Abstract

*Illigera celebica* is an evergreen woody vine that belongs to genus *Illigera* Bl in the family Hernandiaceae and has medicinal value. The complete chloroplast genome of *I. celebica* was sequenced to determine its phylogenetic location with respect to the other species under the Laurales. Its whole chloroplast genome is 156,123 bp in length, and comprises a large single-copy region (LSC, 84,913 bp), a small single-copy region (SSC,18,775 bp), and a pair of inverted repeats (IRs, 26,217 bp). The overall GC content is 39.2% (LSC, 37.8%; SSC, 33.9%; IR, 43.4%). Maximum likelihood phylogenetic analysise (TVM + F + R2 model) was conducted using 15 complete chloroplast genomes of Laurales, and the results confirmed that *Hernandia nymphaeifolia* and *Wilkiea huegeliana* were located in the same lineage.

*Illigera celebica* is a kind of evergreen woody vine that belongs to genus *Illigera* Bl in the family Hernandiaceae. *I. celebica* is widely distributed in Yunnan, Guangxi, and Guangdong provinces (Chinese Flora Editorial Board, Chinese Academy of Sciences 1982) of south China, and in other countries, such as Vietnam, Thailand, and Cambodia (http://foc.iplant.cn/). The root and stem of *I. celebica* can dispel wind, dehumidify, and relieve pain (Huang 1985; Gao 2007). At present, genus *Illigera* has no clear phylogenetic system. Therefore, the complete chloroplast genome of *I. celebica* was obtained by high-throughput sequencing to reconstruct a phylogenetic tree to better understand the relationships of *I. celebica* and other Laurales species.

The healthy young leaves of *I. celebica* were freshly picked from Xishuangbanna Tropical Botanical Garden (XTBG) in Yunnan, China (101.2713°E longitude, 21.9170°N latitude; 540 m). DNA was extracted using modified CTBA method (Cai et al. 2014) and the specimens were stored in XTBG’s Biodiversity Research Group (Registry No. SWFU-SY36764). The whole chloroplast genome was sequenced according to the method of Yang et al. (2014). The whole nine pairs of universal primers were sequenced by remote polymerase chain reaction for next-generation sequencing. The publicly available chloroplast genome of *Eusideroxylon zwageri* (Accession No.MF939351) was used as reference. The chloroplast genome of *I. celebica* was assembled using the GetOrganelle software (Jin et al. 2018) and annotated through the Geneious 8.1.3 software (Biomatters Ltd., Auckland, New Zealand).

The chloroplast genome of *I. celebica* (LAU00199) with a length of 156,123bp, which is 1,639 and 1,454 bp smaller than those of *Hernandia nymphaeifolia* (157,762 bp, MG838431) and *E. zwageri* (157,577 bp, MF939351). It was also 22,963 bp large than that of *Wilkiea huegeliana* (133,160 bp, KT716505). The results showed that the complete genome of *I. celebica* is composed of a large single-copy region (LSC, 84,913 bp), a small single-copy region (SSC, 18,775 bp), and a pair of inverted repeats (IRs, 26,217 bp). The overall GC content is 39.2% (LSC, 37.8%; SSC, 33.9%; IR, 43.4%). The chloroplast genome of *I. celebica* contains 112 unique genes, which are composed of 76 protein-coding genes, 8 are rRNA genes, and 37 tRNA genes.

The evolutionary relationship between *I. celebica* and other Laurales species was determined based on the complete sequence of the chloroplast genome of *I. celebica* and the reconstruction of a phylogenetic tree from the thirteen published chloroplast genomes of family Lauraceae (Figure 1). In addition, *Liriodendron chinense* (Accession Number: KU170538) was treated as an outgroup. A maximum-likelihood (ML) analysis based on the TVM + F + R2 model was performed with iqtree version 1.6.7 program using 1000 bootstrap replicates (Nguyen et al. 2015). The ML phylogenetic tree of *Liriodendron chinense* showed 100% bootstrap values at each node, confirmed that *H. nymphaeifolia* and *Wilkiea huegeliana* belong to the same lineage (Song et al. 2019).

**Figure 1.**
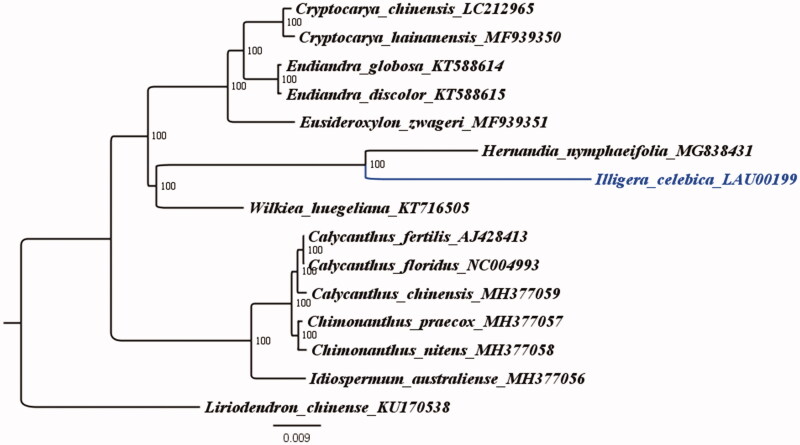
The ML phylogenetic tree for *I. celebica* based on other 14 species (two in *Cryptocarya*, two in *Endiandra*, one in *Eusideroxylon*, one in *Hernandia*, one in *Wilkiea*, three in *Calycanthus*, two in *Chimonanthus*, and one in *Idiospermum*) chloroplast genomes.

## Data Availability

The chloroplast data of the *I. celebica* will be submitted to Laurales Chloroplast Genome Database (https://lcgdb.wordpress.com). Accession numbers are LAU00199.
